# A Systematic Review of the Usage of Rotigotine During Early and Advanced Stage Parkinson's

**DOI:** 10.7759/cureus.36211

**Published:** 2023-03-15

**Authors:** Avanthika Rajendran, Akshay J Reddy, Karol Bisaga, Dillon A Sommer, Neha Prakash, Vivek T Pokala, Zeyu Yu, Mark Bachir, Neel Nawathey, Telak Brahmbhatt, Rakesh Patel

**Affiliations:** 1 Neuroscience, Reed College, Portland, USA; 2 Medicine, California University of Science and Medicine, Colton, USA; 3 Medicine, Midwestern University Chicago College of Osteopathic Medicine, Chicago, USA; 4 Medicine, A.T. Still University, Osteopathic Medical School, Mesa, USA; 5 Health Sciences, California Northstate University, Rancho Cordova, USA; 6 Internal Medicine, East Tennessee State University Quillen College of Medicine, Johnson City, USA

**Keywords:** parkinson's disease, motor function, motor delay, levodopa, rotigotine

## Abstract

Parkinson's disease (PD) is a prevalent neurodegenerative disorder that occurs in old age due to a decrease in dopamine, which causes nerve cell destruction. This disease is difficult to diagnose since its symptoms are similar to those of the aging process. Those with PD have impaired motor control and function, dyskinesia, and tremors. To treat PD, drugs that enhance the amount of dopamine given to the brain are administered to alleviate symptoms. This inquiry examines the prescription of rotigotine to achieve this objective. The primary objective of this review is to examine the usage of rotigotine in both the late and early stages of PD. The statistical model utilized in the review found that there was not a significant difference in the dosage of rotigotine prescribed to late and early-stage PD patients, however, there were some confounding variables that may have skewed this result; therefore, further research is necessary to validate or nullify this hypothesis.

## Introduction and background

Parkinson’s disease (PD) is a neurological disorder that leads to shaking, difficulty with balance, and coordination [1]. When Parkinson's symptoms appear, they gradually worsen over time and as the disease progresses, patients have difficulty in their motor functions and talking. This condition is caused when nerve cells in the basal ganglia become impaired or die. When those neurons become impaired or die, there is a decrease in dopamine production that leads to movement problems associated with PD [2]. The rate of PD progression differs among patients because some of the symptoms associated with it are symptoms of normal aging, and without medical tests to properly diagnose the disease, it can be difficult to accurately diagnose. PD most commonly appears in patients in ages 60 and older; researchers aren’t sure if it is solely a genetic mutation or hereditary, but more often than not, it happens randomly [3]. There is currently no cure for PD, but there are a few medications and therapies in place to relieve symptoms. Drugs that are prescribed for PD are meant to increase the level of dopamine in the brain and help control motor and non-motor symptoms. The most common therapy for PD is levodopa where the nerve cells use levodopa to have dopamine replenish the brain’s decreasing supply of it [4-5]. A newer PD treatment that has been prescribed more is the rotigotine transdermal patch, which is a dopamine agonist that restores the balance of dopamine in the brain. Dopamine agonists mimic the effects of dopamine in the brain; it is not as effective as levodopa when looking at muscle movement and rigidity [1]. This medication helps motor movement, which decreases tremors, stiffness, and unsteadiness.

## Review

Methods

**Figure 1:**
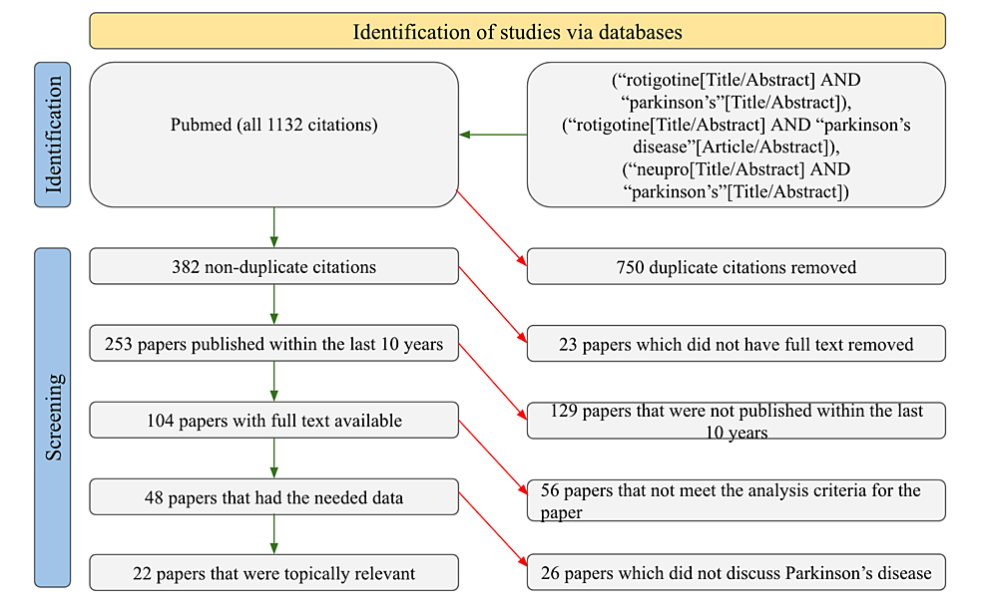
PRISMA diagram PRISMA: Preferred Reporting Items for Systematic Reviews and Meta-Analyses

Searches were conducted on PubMed to find studies regarding the use of rotigotine and Parkinson's disease. Exact searches were done with the keywords “rotigotine and parkinson’s”. All time frames were searched, which brought a pool of 382 studies. Of those studies, 253 were published in the last 10 years, 104 mentioned the length of the study, 85 mentioned the sample sizes, 48 provided dosage amounts, and only 22 had the stage of Parkinson’s disease listed. The data collected from these 22 studies included the dosage of rotigotine that was used, the sample size of patients in each study, the majority category of PD stage in those patients, the length of the study, and the combination agents used with rotigotine. Studies that did not report enough information to overlap with at least two of these categories were excluded in order to remove potential personal bias. Figure 1 provides a clearer illustration of the filtering procedure used by the authors of this review.

Diagnosis of Parkinson's disease

Unfortunately, a PD diagnosis often occurs after symptoms begin. Diagnosis occurs by conducting a patient neurological examination and a review of symptoms, looking for the four cardinal symptoms of PD: tremors in extremities, muscle stiffness, slowness of movement, and impaired balance and coordination [3-5]. Imaging such as computerized tomography or magnetic resonance imaging can aid in the diagnosis but is never the only factor determining a diagnosis [3-6]. Symptoms generally develop gradually and worsen over time, and therefore, it may be difficult for a physician to catch the early stages of PD in patients with an unknown baseline of behavior and diagnosis may not be completed until the PD has progressed to an advanced stage. Additionally, as PD involves the loss of the substantia nigra of the brain, a tissue that generates dopamine for the body, there is no cure for the disease [5]. Treatment, therefore, takes the form of introducing dopamine into the brain, inhibiting dopamine catabolism, and controlling symptoms. Rotigotine, a transdermal patch dopamine agonist treatment, works as a partial or full agonist of an array of dopamine receptors, restoring the levels of effective dopamine in the brain. Other treatments for PD often aim to stimulate similar pathways or increase dopamine in the brain. One such treatment includes levodopa, a chemical precursor to dopamine that unlike dopamine can cross the blood-brain barrier [6-12]. Prolonged use of levodopa often leads to complications such as PD fluctuation of symptoms and problems with involuntary movements, opening the doorway to combining levodopa treatment with other treatments to combat side effects. The studies analyzed in Table 1 found that advanced-stage PD was more frequently detected and more present in groups with larger sample sizes, suggesting that early-stage PD may be difficult to detect, thereby leading to fewer studies with patients with early-stage PD, as early-stage PD is characterized by subtle changes in patient behavior, including changes in motor function and coordination that may be difficult to recognize until they develop further [6-27]. This means that advanced-stage PD may often be detected more frequently than early-stage PD, as the development of the disease worsens all of the symptoms of the disease in its early stage (advanced motor fluctuation and dyskinesia), in addition to introducing new ones (marked cognitive decline, speech changes, dementia, fecal incontinence, and so forth) and are noticeable even to non-professionals. Statistical analysis was done in Tables 2-3 by calculating means and then by employing a two-paired t-test.

**Table 1:** An analysis of rotigotine usage for the treatment of Parkinson’s disease

Author (year)	Rotigotine Dosage	Sample Size	Majority Category of Parkinson’s Stage	Combination Agents	Length of Study
Chaudhuri (2013) [6]	2-16 mg/24 hr	287	Advanced stage	Levodopa	8 weeks
Ferrazzoli (2018) [7]	2 mg/24 hr and increased to 4mg/24h after 7 days	36	Early stage	None	18 months
Sieb (2015) [8]	6 mg/24 hr	147	Advanced stage	Levodopa	one month
Cawello (2014) [9]	2 mg/24 hr	48	Both	Levodopa	2 weeks
Isaacson (2019) [10]	4.8-3.9 mg/24 hr	39	Early stage	Levodopa	12 weeks
Zhang (2017) [11]	4-16 mg/24 hr	346	Advanced Stage	Levodopa	12 weeks
Kim (2015) [12]	went from 2mg/24 hr to 4 mg/24 hr	48	Advanced Stage	Levodopa	9 days
Nomoto (2014) [13]	16 mg/24 hr	174	Advanced Stage	Levodopa	19 weeks
Hauser (2016) [14]	2-16 mg/24 hr	122	Early Stage	Levodopa / None	12 weeks
Nomoto (2017) [15]	8-16 mg/24 hr	2057	Advanced Stage	Levodopa	12 weeks
Giladi (2013) [16]	16 mg/24 hr	381	Early Stage	Levodopa	6 years
LeWitt (2012) [17]	16 mg/24 hr	656	Early Stage	Levodopa	6 years
Woitalla (2018) [18]	4.9 - 6.1 mg/24 hr 6 months	70	Both	Levodopa	6 month
Chitnis (2012) [19]	6 mg/24 hr	33	N/A	Levodopa	3 months
Antonini (2015) [20]	8mg/24 hr to 16 mg/24 hr	249	Both	Monoamine oxidase B inhibitor	12 weeks
Kim (2015) [21]	8mg/24 hr	90	Early Stage	Levodopa	8 weeks
Bhidayasiri (2017) [22]	10.46 mg/24 hr	34	Both	Budipine	8 weeks
Chung (2016) [23]	low dose: 6 mg/24 hr for early PD and 8mg/24 hr for advanced PD high dose: 8 mg/24 hrs for early PD and 16 mg/24 hr for advanced PD	380	Both	Levodopa	8 weeks
LeWitt (2016) [24]	switched to rotigotine with 14 mg/24 hr	87	Both	Levodopa	4 weeks
Giladi (2016) [25]	8mg/24 hr	596	Both	Levodopa	Had two separate groups that were 5 years and 3 months and 4 years and 3 month
Rascol (2015) [26]	16 mg/24 hr	68	Advanced Stage	Levodopa	12 weeks
Moretti (2014) [27]	3.3 mg/ 24 hr	61	Both	Levodopa	1 year

**Table 2:** Statistical analysis using t-test and p-values for rotigotine dosage between early and advanced-stage Parkinson’s patients

Group Comparison	t-value	p-value
Rotigotine Dosage in Early vs Advanced Stage PD	0.71896	0.47715

**Table 3:** Monoamine vs. levodopa

Group comparison	t-value	p-value
Levodopa vs. Non-Levodopa	0.0317	0.9747
Monoamine vs. Non-Levodopa	0.0277	.9779

Given the significant range and severity of symptoms between the early and advanced stages of PD, we would assume the administered doses of transdermal rotigotine to each group would vary as well. The mean dosage of rotigotine used in early-stage PD patients was calculated to be 9.25 mg/24 hr while the advanced-stage dosage of rotigotine was reported to be 11.71 mg/24 hr. However, according to the t-test and p-values provided in Table 2, when comparing the dosages administered to early and advanced-stage PD patients there was no significant difference between the two groups. Therefore further investigation should be conducted before ascertaining whether or not there is a variance in the dosage of rotigotine that is administered to advanced and early PD patients. This analysis was performed by using the average rotigotine dosage amounts administered to each group from the articles that were compiled from the literature review. In order to improve outcomes for PD patients, perhaps rotigotine dosage amounts could be a point of investigation given that an increase in dosage is recommended for patients with more advanced stage PD.

Levodopa usage in the treatment of Parkinson’s disease

Levodopa is the most frequently prescribed drug for PD treatment, as it can cross the blood-brain barrier and be metabolized by the brain into dopamine, which can replenish the brain’s supply after losing its own ability to create sufficient dopamine. Levodopa functions by entering the patient’s blood and crossing into the brain via the blood-brain barrier; however, only 5-10% of administered levodopa enters this pathway [23-25]. The rest of the administered levodopa enters the systemic circulation and can be deposited into other tissues, where it may be metabolized into dopamine, causing side effects including nausea and vomiting [6,7]. The metabolism of levodopa into dopamine in tissues other than the brain is therefore controlled by dopa decarboxylase inhibitors, which inhibit this pathway; these drugs include carbidopa and benserazide. In addition to symptoms that may arise from incomplete inhibition of the metabolism of levodopa into dopamine in the peripheral tissues, prolonged levodopa use often results in dyskinesia due to a possible DNA methylation mechanism that increases the brain’s sensitivity to levodopa that can only be stopped by halting levodopa treatment altogether [15-19]. To combat this increased sensitivity, levodopa is often administered in smaller doses and in combination with other treatments, including other dopamine agonists, such as rotigotine, which can act as functional dopamine in the brain and lessen the sensitivity developed over time from levodopa use. In the studies reviewed, levodopa was the most frequent combination drug used alongside rotigotine, though other combinations included monoamine oxidase B inhibitor, a dopamine breakdown inhibitor, and budipine, a dopamine synthesis promoter [17-18].

The clinical use case of this review is finding whether patients required higher dosages of rotigotine in combination with levodopa or other combination drugs and whether the stage of PD required higher doses of rotigotine. By conducting a statistical analysis for a significant difference in these two pairs of categories, this study can contribute to a physician’s determination of the optimal rotigotine dosage to treat a patient after gathering information about the progression of their disease and which other combination drugs they will be prescribed. The results of this analysis are displayed in Table 3. It was found that patients taking rotigotine in combination with levodopa were prescribed on average a levodopa dosage of 9.22 mg/24 hr. Patients that were taking rotigotine in combination with drugs other than levodopa were prescribed on average a levodopa dosage of 8.89 mg/24hr, and the difference in dosages between the two groups was found to be not statistically significant (p = 0.97). Additionally, the differences in dosages of rotigotine in milligrams were found to be insignificant for monoamine combinations versus non-levodopa combinations (p = 0.98), and, finally, the difference in average dosages in milligrams of rotigotine for early PD patients versus advanced stage PD patients was also found to be statistically insignificant (p = 0.48). These results likely indicate that rotigotine dosage size should not necessarily change for patients with early-stage PD versus advanced-stage PD, as well as for levodopa versus non-levodopa combination regimens and monoamine versus non-levodopa combination regimens.

Current limitations and future applications

The limitations of this study include limitations to the statistical analysis, which include limited sample size and several assumptions built into the usage of a paired t-test, which includes assumptions of the dosage sizes being normally distributed and that its variance among all the samples is constant and that the sample of PD patients chosen is representative of the total population of PD patients; because of this, the results suggested by the statistical analysis may be inaccurate. Non-statistical limitations include not tracking the average treatment outcomes of patients with higher rotigotine dosages among early-stage and advanced-stage PD patients, as well as having more advanced-stage PD patients represented in the fielded sample than early-stage PD patients. Additionally, there were far more samples of levodopa combination treatments than rotigotine/monoamine combination treatments in the fielded sample population. Finally, the fielded studies varied greatly in time length, from nine days to six years in duration, possibly having an effect on the results of this analysis. Information about the administration route of rotigotine or the other combination drugs would also be useful in determining possible differences in treatment effects. Further investigations into the treatment outcomes per size of rotigotine dosage would allow for stronger conclusions to be made about whether advanced-stage PD patients require higher doses of rotigotine for effective treatment or vice versa, which could contribute to further insights into the mechanism of PD. Additionally, differences in dosages for effective treatment in combination with other drugs, including levodopa, monoamine oxidase B inhibitor, and budipine, could help gather insight into these drugs' biochemical mechanisms, as well as any potential drug interactions. Finally, conducting further review of available rotigotine treatment data (possibly through consulting foreign language journals or otherwise expanding the sources of data on patients with PD) could yield larger sample sizes, which would increase confidence in any statistical conclusions made, as well as possibly drive insights into other factors that may drive differences in treatment efficacy, including differences in rotigotine efficacy in sex, age, ethnicity, or other potential categories. Differences in rotigotine efficacy or PD symptoms in different population demographics are important to clarify in future investigations, as they may undermine the assumptions behind the statistical analysis, which include samples that represent the overall population and the assumption of constant variance.

## Conclusions

The treatment of Parkinson's disease involves supplying patients with medications that function as dopamine agonists. This paper focuses on analyzing how rotigotine, a specific dopamine agonist, is used in conjunction with other medications in the treatment of both advanced and early-stage Parkinson's patients. After conducting a statistical analysis using data from the selected articles within the review, it was found that when different combination agents were used in the treatment of Parkinson's disease, the dosage of rotigotine prescribed did not significantly change. Additionally, the dosage of rotigotine was not found to be significantly different when being used to treat late and early-stage Parkinson's patients. Further investigation is needed to understand how rotigotine dosage affects long-term patient outcomes.
